# Geriatrie und geriatrische Aspekte im Koalitionsvertrag 2021

**DOI:** 10.1007/s00391-023-02226-8

**Published:** 2023-09-03

**Authors:** Pascal Nohl-Deryk, Stefan Grund

**Affiliations:** 1 Institut für Allgemeinmedizin, Uniklinik Köln, Kerpener Straße 62, 50937 Köln, Deutschland; 2 Agaplesion Bethanien Krankenhaus Heidelberg, Rohrbacher Straße 149, 69126 Heidelberg, Deutschland

**Keywords:** Demografische Entwicklung, Barrierefreiheit, Medizinische Versorgung, Rehabilitation, Altenpflege, Demographic change, Accessibility, Medical care, Rehabilitation, Geriatric nursing

## Abstract

**Hintergrund:**

Der Anteil älterer Menschen in Deutschland wächst stark. Bis zur Bundestagswahl 2025 geht das Statistische Bundesamt davon aus, dass ein Drittel der Bevölkerung das Rentenalter erreicht haben wird. Ältere Menschen sind damit eine der größten Wählergruppen und politisch besonders relevant.

**Fragestellung:**

Wie fördert die Bundesregierung die Lebensbedingungen dieser Wählergruppe? Was plant sie hinsichtlich der medizinischen Versorgung älterer Menschen?

**Methoden:**

Es erfolgte eine Inhaltsanalyse des Koalitionsvertrages nach Aspekten demografischer Entwicklung, geriatrischer Versorgung und Lebensbedingungen älterer Menschen. Die kodierten Textstellen wurden gemischt induktiv-deduktiv kategorisiert. Schließlich wurde der Umsetzungsfortschritt konkreter Vorhaben nach eineinhalb Jahren Amtszeit beurteilt.

**Ergebnisse:**

Im Text des Koalitionsvertrages wurden 23 Textstellen mit knapp 620 Wörtern (gut 1 % des Textes) identifiziert und kategorisiert. Neun Textstellen ließen sich dem Themenbereich „ältere Bevölkerung“, 6 Textstellen der „medizinischen Versorgung“ und 8 der „pflegerischen Versorgung“ zuordnen. 14 von 23 Textstellen enthielten konkrete Handlungsvorhaben oder Zieldaten. In den ersten eineinhalb Jahren der Bundesregierung konnte bei 4 davon ein teilweiser Fortschritt identifiziert werden, eine Umsetzung erfolgte bei keinem Vorhaben.

**Diskussion:**

Der Koalitionsvertrag lässt insgesamt ein Bewusstsein für eine älter werdende Bevölkerung erkennen. In Anbetracht der demografischen Entwicklung werden allerdings wenige Lösungsvorschläge für genuin geriatrische Aspekte gemacht. Das erste Amtsjahr war stark durch andere Themen geprägt, woraus sich ungeplante Gesetze und Verordnungen ergaben und Vorhaben aus dem Koalitionsvertrag in den Hintergrund rückten.

## Abstract (en)

**Background:**

The proportion of older people in Germany is increasing. By the next federal election in 2025 the Federal Statistical Office of Germany expects that one third of the population will have reached retirement age. Older people are thus one of the largest voter groups and are particularly politically relevant.

**Objective:**

How does the federal government support the living conditions of this voter group? What are the plans of the federal government regarding medical care of older people?

**Methods:**

A content analysis of the coalition agreement was carried out with respect to aspects of demographic change, geriatric care and living conditions of older people. The coded text passages were categorized using a mixed inductive-deductive approach. Finally, the implementation progress of concrete projects after 1.5 years in office was evaluated.

**Results:**

This study identified and categorized 23 passages with just under 620 words (about 1% of the total text) in the coalition agreement. Of the passages nine were assigned to the topic older population, six passages to medical care and eight to nursing care. Of the 23 passages 14 contained concrete plans for action or target dates. In the first 1.5 years of the federal government, partial progress could be identified in 4 of these, while no projects were realized.

**Conclusion:**

Overall, the coalition agreement shows an awareness for an ageing population. In view of the demographic development, however, few solutions are proposed for genuine geriatric aspects. The first year in office was strongly influenced by other topics, which resulted in unplanned laws and regulations and projects from the coalition agreement faded into the background.

## Hintergrund und Fragestellung

Ende des Jahres 2020 lebten in Deutschland 83,1 Mio. Menschen. Davon waren 28,9 % über 60 Jahre alt. Seit der letzten Bundestagswahl im Jahr 2017 ist die Anzahl der über 60-Jährigen in Deutschland um über 1 Mio. Menschen gewachsen und der Bevölkerungsanteil um einen Prozentpunkt gestiegen. Besonders die Subgruppe der über 80-Jährigen wächst rasch [9]. Das Statistische Bundesamt geht bis 2035 von einem erheblichen Zuwachs der älteren Bevölkerungsanteile aus und rechnet bis zur nächsten Bundestagswahl 2025 damit, dass über ein Drittel der Bevölkerung das Rentenalter erreicht haben wird [8].

Für die Bundestagswahl 2021 waren alle volljährigen, also über 18-jährigen, Deutschen zur Wahl aufgerufen. Der Anteil der über 60-Jährigen an den Wahlberechtigten lag bei über 38 %, von welchen 77,4 % gewählt haben und damit 39 % aller abgegeben Stimmen ausmachen [4]. Sofern diese heterogene Gruppe unter dem Terminus „ältere Menschen“ zusammengefasst wird, ist sie durchaus eine der größten, wenn nicht sogar die größte Wählergruppe. Zu ihr gehören Menschen, die kurz vor oder im Ruhestand sind, in einem Senioren- oder Pflegeheim oder zu Hause alt werden. Auch im Gesundheitswesen macht diese Personengruppe einen großen Teil der vollstationären Patientinnen und Patienten aus und führt zu vielen Behandlungsanlässen im ambulanten Sektor.

Der ältere Teil der Bevölkerung kann also wahlentscheidend sein und wird künftig noch relevanter. Wie fördert also die neue Bundesregierung die Lebensbedingungen dieser Wählergruppe? Was plant sie hinsichtlich der medizinischen Versorgung der größten Patientengruppe?

Welche Ziele und Maßnahmen die neue Bundesregierung aus Sozialdemokratischer Partei Deutschlands (SPD), Bündnis 90/Die Grünen (Grüne) und Freiheitlicher Demokratischer Partei (FDP) in ihrem am 07.12.2021 unterzeichneten Koalitionsvertrag [11] zu diesen Fragestellungen festgehalten hat, soll der nachfolgende Beitrag skizzieren. Dazu erfolgt eine Analyse des Vertrages hinsichtlich verschiedener Aspekte zum Leben älterer Menschen und deren gesundheitlicher Versorgung. Zusätzlich erfolgt eine Betrachtung, welche der Vorhaben im ersten Jahr nach der Vereidigung umgesetzt oder angestoßen wurden.

## Methodik

Für die Analyse des Koalitionsvertrages wurde ein inhaltsanalytischer Ansatz gewählt und methodisch an die Arbeit einer der Autoren zum Koalitionsvertrag 2018 angelehnt [7]. Einer der Autoren analysierte den Koalitionsvertrag vollständig und kodierte alle Textstellen, die sich folgenden Feldern zuordnen ließen:

- demografische Entwicklung,
- medizinische Versorgung älterer Menschen, einschließlich ambulanter, stationärer und rehabilitativer Versorgung,
- Altenpflege, einschließlich ambulanter Pflege und nichtprofessioneller Pflege älterer Menschen,
- Lebensbedingungen älterer Menschen, beispielsweise zur Barrierefreiheit oder zu Wohnformen.

Ein weiterer Autor glich alle kodierten Textstellen mit dem Originaltext ab und prüfte stichprobenartig die Vollständigkeit der Kodierung. Anschließend erfolgte eine gemischt induktive-deduktive Einordung der Textstellen in ein Kategoriensystem, um die für die Geriatrie/Gerontologie relevanten Inhalte thematisch zu strukturieren. Die deduktive Einordnung erfolgte dabei anhand der Wahlprüfsteine Gesundheit zur Bundestagswahl 2021 der Bundesarbeitsgemeinschaft der Seniorenorganisationen (BAGSO) [12]. Die qualitative Inhaltsanalyse wurde mit Dedoose durchgeführt.

Zur Betrachtung des Umsetzungsfortschritts wurde der Zeitraum vom 08.12.2021, dem Tag der Vereidigung der neuen Bundesregierung, bis zum 07.05.2023 gewählt. Aus den kodierten Textstellen wurden jene mit einer konkreten Handlungsabsicht oder einem Zieldatum für eine Analyse des Umsetzungsfortschritts ausgewählt. Es erfolgte eine manuelle Recherche der Webseiten der Bundesregierung, der zuständigen Bundesministerien und des *Gesetzgebungskalender Gesundheitspolitik* des AOK-Bundesverbands. Bei Ergebnislosigkeit der ersten Recherche wurde eine Suchmaschinensuche gestartet, und die ersten 30 Ergebnisse wurden auf Hinweise ausgewertet.

## Ergebnisse

Der Koalitionsvertrag umfasst 178 Seiten und knapp 52.000 Wörter. Im gesamten Text wurden nach oben beschriebenem Vorgehen insgesamt 23 Textstellen mit knapp 620 Wörtern, also gut 1 % des Textes, identifiziert und dann thematisch neu geordnet (Tab. 1). Unter den 3 alphabetisch sortierten Oberthemen ältere Bevölkerung, medizinische Versorgung und pflegerische Versorgung ließen sich alle Kodierungen einordnen. Dabei entfielen 9 Textstellen primär auf den Themenbereich ältere Bevölkerung, 6 auf medizinische Versorgung und 8 auf pflegerische Versorgung.

**Table 1:** 

Thema	Auszüge	Seite
*Ältere Bevölkerung*		
Demografischer Wandel	Unsere Gesellschaft wird älter und diverser	4
	Wir wollen selbstbestimmtes Leben für ältere Menschen unterstützen und den Zusammenhalt zwischen den Generationen fördern	98
	Erfahrungen und Kompetenzen älterer Menschen sind für unsere Gesellschaft unverzichtbar	102
Selbstbestimmtes Leben	Wir wollen, dass Menschen im Alter selbstbestimmt in ihrem frei gewählten Umfeld leben können	102
	Wir werden seniorengerechte Ansätze auf allen staatlichen Ebenen und im digitalen Raum fördern. Dabei geht es u. a. um Partizipation, Engagement, soziale Sicherung, Alltagshilfen, Wohnen, Mobilität, Gesundheitsvorsorge, Bildungs- und Begegnungsangebote und die Überwindung von Einsamkeit	102
	Wir werden ältere Menschen vor Diskriminierung und vor finanzieller Ausbeutung – insb. durch Vorsorgevollmachten – schützen	103
	Wir begrüßen, wenn durch zeitnahe fraktionsübergreifende Anträge das Thema Sterbehilfe einer Entscheidung zugeführt wird	113
Barrierefreiheit	Wir wollen, dass Deutschland in allen Bereichen des öffentlichen und privaten Lebens, vor allem aber bei der Mobilität (u. a. bei der Deutschen Bahn), beim Wohnen, in der Gesundheit und im digitalen Bereich, barrierefrei wird. Wir setzen dafür das Bundesprogramm Barrierefreiheit ein. […] Wir setzen uns das Ziel, alle öffentlichen Gebäude des Bundes umfassend barrierefrei zu machen. Wir verpflichten in dieser Wahlperiode private Anbieter von Gütern und Dienstleistungen innerhalb einer angemessenen Übergangsfrist zum Abbau von Barrieren oder, sofern dies nicht möglich oder zumutbar ist, zum Ergreifen angemessener Vorkehrungen. Wir legen entsprechende Förderprogramme auf und bauen die Beratungsarbeit der Bundesfachstelle Barrierefreiheit aus	78
	Für ein diverses, inklusives und barrierefreies Gesundheitswesen erarbeiten wir mit den Beteiligten bis Ende 2022 einen Aktionsplan	85
*Medizinische Versorgung*		
	Zentrale Zukunftsfelder sind unter anderem: […] Drittens: ein vorsorgendes, krisenfestes und modernes Gesundheitssystem, welches die Chancen biotechnologischer und medizinischer Verfahren nutzt, und das altersabhängige Erkrankungen sowie seltene oder armutsbedingte Krankheiten bekämpft	20
Rehabilitative Versorgung	Den Zugang zu Maßnahmen der Prävention und Rehabilitation werden wir vereinfachen sowie das Reha-Budget bedarfsgerechter ausgestalten	74
Prävention	Mit einem Klimaanpassungsgesetz schaffen wir einen Rahmen, um gemeinsam mit den Ländern eine nationale Klimaanpassungsstrategie mit messbaren Zielen etwa in den Handlungsfeldern Hitzevorsorge, Gesundheits- und Allergieprävention und Wasserinfrastruktur umzusetzen und rechtzeitig nachsteuern zu können	40
	Wir machen längeres, gesünderes Arbeiten zu einem Schwerpunkt unserer Alterssicherungspolitik	74
	Wir schaffen einen Nationalen Präventionsplan sowie konkrete Maßnahmenpakete z. B. zu den Themen Alterszahngesundheit, Diabetes, Einsamkeit, Suizid, Wiederbelebung und Vorbeugung von klima- und umweltbedingten Gesundheitsschäden	84
Forschung	Wir wollen die vorhandenen Kompetenzen und Entwicklungspotenziale weiter stärken, indem wir mit zusätzlichen Mitteln ein neues Forschungszentrum der Helmholtz-Gemeinschaft für Alternsforschung errichten	21
*Pflegerische Versorgung*		
	Bei der pflegerischen Versorgung vor Ort räumen wir den Kommunen im Rahmen der Versorgungsverträge verbindliche Mitgestaltungsmöglichkeiten ein	81
	Bei der intensivpflegerischen Versorgung muss die freie Wahl des Wohnorts erhalten bleiben. Das Intensivpflege- und Rehabilitationsstärkungsgesetz (IPReG) soll darauf hin evaluiert und nötigenfalls nachgesteuert werden. Wir gestalten eine rechtssichere Grundlage für die 24-Stunden-Betreuung im familiären Bereich	81
	Professionelle Pflege ergänzen wir durch heilkundliche Tätigkeiten und schaffen u. a. das neue Berufsbild der „Community Health Nurse“	82
Pflegende Angehörige	Wir unterstützen den bedarfsgerechten Ausbau der Tages- und Nachtpflege sowie insbesondere der solitären Kurzzeitpflege	81
	Leistungen wie die Kurzzeit- und Verhinderungspflege fassen wir in einem unbürokratischen, transparenten und flexiblen Entlastungsbudget mit Nachweispflicht zusammen, um die häusliche Pflege zu stärken	81
	Wir entwickeln die Pflegezeit- und Familienpflegezeitgesetze weiter und ermöglichen pflegenden Angehörigen und Nahestehenden mehr Zeitsouveränität, auch durch eine Lohnersatzleistung im Falle pflegebedingter Auszeiten	81
Stärkung der Altenpflege	In der stationären Langzeitpflege beschleunigen wir den Ausbau der Personalbemessungsverfahren. Insbesondere dort verbessern wir Löhne und Arbeitsbedingungen der Pflegekräfte mit dem Ziel, die Gehaltslücke zwischen Kranken- und Altenpflege zu schließen	81
Attraktivität der Pflegeberufe	Wir wollen den Pflegeberuf attraktiver machen, etwa mit Steuerbefreiung von Zuschlägen, durch die Abschaffung geteilter Dienste, die Einführung trägereigener Springerpools	81

Aus den 23 Textstellen wurden 14 mit konkreten Handlungsvorhaben oder Zieldaten für eine Betrachtung des Umsetzungsfortschritts ausgewählt. Darunter befand sich ein Punkt mit einem konkreten Zieldatum bis Ende 2022.

In den ersten eineinhalb Jahren der Bundesregierung konnte von den für die Geriatrie/Gerontologie relevanten Punkten bei 4 davon ein teilweiser Fortschritt verzeichnet werden, eine Umsetzung erfolgte noch bei keinem der aufgeführten Vorhaben (Tab. 2). Eine im Koalitionsvertrag bis Ende 2022 vorgesehene Erarbeitung eines Aktionsplans für ein diverses, inklusives und barrierefreies Gesundheitswesen ist nicht erfolgt. Eine Evaluation des Intensivpflege- und Rehabilitationsstärkungsgesetz (IPReG) konnte, aufgrund noch laufender Verhandlungen bis zum Ende des betrachteten Zeitraums, noch nicht erfolgen.

**Table 2:** 

Thema	Zusammenfassung der Auszüge	Umsetzung
*Ältere Bevölkerung*		
Selbstbestimmtes Leben	Sterbehilfe: zeitnahe Entscheidung	Es ist eine fraktionsübergreifende Debatte und Abstimmung vor der Sommerpause vorgesehen. Zwei interfraktionelle Gesetzentwürfe liegen vor
Barrierefreiheit	Barrierefreiheit; Bundesprogramm Barrierefreiheit; Ausbau der Bundesfachstelle	Im ersten Jahr keine Umsetzung
	Aktionsplan diverses, inklusives und barrierefreies Gesundheitswesen bis Ende 2022	Ein Aktionsplan wurde nicht bis Ende 2022 erarbeitet
*Medizinische Versorgung*		
Rehabilitative Versorgung	Zugang zu Rehabilitation vereinfachen; bedarfsgerechtes Reha-Budget	Im ersten Jahr keine Umsetzung. Es wurde ein Hilfsfonds zur Unterstützung bei gestiegenen Energiekosten, auch für Reha-Einrichtungen, aufgesetzt
Prävention	Klimaanpassungsgesetz, u. a. zu Hitzevorsorge, Gesundheits- und Allergieprävention	Ein Referentenentwurf wurde im April vorgestellt. Federführend ist das Bundesumweltministerium
	Nationaler Präventionsplan und Einzelmaßnahmen	Im ersten Jahr keine Umsetzung
Forschung	Helmholtz-Forschungszentrum für Alternsforschung	Mainz wurde als Standort festgelegt. Erste Konzepte werden erstellt
*Pflegerische Versorgung*		
	Mitgestaltungsmöglichkeiten der Kommunen bei der pflegerischen Versorgung	Im ersten Jahr keine Umsetzung
	Intensivpflegerische Versorgung; Evaluation des Intensivpflege- und Rehabilitationsstärkungsgesetzes (IPReG); Rechtsgrundlage für 24-h-Betreuung	Bis Ende 2022 erfolgten noch Vertragsverhandlungen zwischen Krankenkassen und Leistungserbringern. Eine Evaluation konnte daher im ersten Jahr nicht erfolgen
	Heilkundliche Tätigkeiten; „Community Health Nurse“	Im ersten Jahr keine Umsetzung
Pflegende Angehörige	Zusammenfassung von Kurzzeit- und Verhinderungspflege in ein Entlastungsbudget	Im ersten Jahr keine Umsetzung
	Weiterentwicklung der Pflegezeit- und Familienpflegezeitgesetze	Im ersten Jahr keine Umsetzung
Stärkung der Altenpflege	Ausbau Personalbemessungsverfahren in Langzeitpflege; Verbesserung Löhne und Arbeitsbedingungen	Das Krankenhauspflegeentlastungsgesetz (KHPflEG) wurde verabschiedet. Darin wird für die stationäre Krankenversorgung die PPR 2.0 als Personalbemessungsverfahren kurzfristig eingeführt
Attraktivität der Pflegeberufe	Steuerbefreiung von Zuschlägen; Abschaffung geteilter Dienste; Einführung trägereigener Springerpools	Im ersten Jahr keine Umsetzung

Der vollständige Auszug aus dem Koalitionsvertrag findet sich jeweils in der vorangegangenen Tab. 1

## Diskussion

Insgesamt lässt sich im Koalitionsvertrag ein Bewusstsein für eine älter werdende Bevölkerung und damit verbundene politische Handlungsfelder erkennen. Zwar umfassen die identifizierten Textstellen nur etwa 1 % des Gesamttextes, die Auszüge sind aber von S. 4 bis S. 113 gestreut und deuten damit auf eine querschnittliche Betrachtung des Themenkomplexes hin.

Nicht alle Auszüge beziehen sich explizit auf geriatrische Aspekte. So kommt Barrierefreiheit v. a. behinderten Menschen, aber auch Personen mit Einschränkungen ohne Grad der Behinderung zugute. Jedoch profitieren gerade auf Hilfsmittel angewiesene Hochbetagte sowohl von einer Barrierefreiheit im öffentlichen Raum wie auch von einem barrierefreien Gesundheitswesen, welches sich positiv auf ihre Teilhabe am gesellschaftlichen Leben auswirken kann.

Der Koalitionsvertrag legt einen großen Fokus auf selbstbestimmtes Leben. Dafür sollen seniorengerechte Ansätze gefördert werden, hierbei wird auch explizit die Gesundheitsvorsorge genannt. Aber auch weitere Aspekte wie Alltagshilfen oder Überwindung von Einsamkeit sind in Bezug auf Morbidität relevant [6].

In Bezug auf die medizinische Versorgung werden altersabhängige Erkrankungen als einige von wenigen zentralen Zukunftsfeldern benannt und damit die Wichtigkeit der Geriatrie erkannt. Weitere medizinische Aspekte mit geriatrischem Bezug, die in einem nationalen Präventionsplan aufgegriffen werden sollen, sind die Alterszahngesundheit, die schon oben genannte Einsamkeit sowie klimabedingte Gesundheitsschäden. Ein anderer Auszug verspricht eine Klimaanpassungsstrategie u. a. zur Hitzevorsorge, die besonders für ältere Personen relevant ist [1]. Im Zuge der Herausforderungen der geriatrischen Rehabilitation wird sich zeigen, inwiefern das Ziel, das Reha-Budget bedarfsgerechter auszugestalten, auch Bezug auf die geriatrische Rehabilitation hat [3].

Interessant ist auch die Ankündigung, ein neues Helmholtz-Forschungszentrum für Alternsforschung aufzubauen. Dabei wird von der konkreten Ausgestaltung abhängen, welchen Anteil auch alltagsrelevante geriatrische Fragestellungen einnehmen, da erste Aussagen auf eher molekularbiologische Forschungsansätze schließen lassen [10].

Der Abschnitt zu Pflege im Koalitionsvertrag ist der erste und auch insgesamt zweitlängste. In der Überschrift des Kapitels heißt es explizit „Pflege und Gesundheit“, wo es im Koalitionsvertrag der Vorgängerregierung noch „Gesundheit und Pflege“ geheißen hatte [13]. Ein Teil der angekündigten Maßnahmen hat vor dem Hintergrund des Personalmangels in der Pflege zum Ziel, das Berufsfeld generell zu stärken und attraktiver zu machen. Die stationäre Langzeitpflege wird explizit thematisiert, und auch hier zielen Maßnahmen auf die Steigerung der Attraktivität des Berufes.

Besonders ausgeprägt werden Maßnahmen zur Unterstützung pflegender Angehöriger ausgeführt, der heute bereits größten Gruppe Pflegender [14]. Dabei werden sowohl unterstützende Strukturen wie die Tages- oder Kurzzeitpflege erwähnt, als auch Werkzeuge zur Unterstützung der Vereinbarkeit von Pflege und Beruf durch Pflegezeit und Verhinderungspflege.

Zur deduktiven Kategorienbildung wurden die Wahlprüfsteine Gesundheit der BAGSO als Referenz verwendet [12]. Die BAGSO stellte den Parteien dabei insgesamt 8 Fragen, u. a. zu ambulanter Rehabilitation, präventivem Hausbesuch, Versorgung von Menschen mit Demenz oder Community Health Nurses. Die erste Frage bezog sich auf geplante Maßnahmen zur Stärkung von Prävention und Rehabilitation für Ältere, diese Punkte werden, zumindest themenbezogen, s. Alterszahngesundheit, im Koalitionsvertrag aufgegriffen.

Die ambulante Rehabilitation wie auch der präventive Hausbesuch findet im Koalitionsvertrag keine explizite Erwähnung. Präventive Ansätze zur Gesunderhaltung Älterer finden sich, von Ausnahmen abgesehen, im Koalitionsvertrag leider kaum.

Eine weitere Frage bezog sich auf die Versorgung von Menschen mit Demenz, dazu gibt es keine Anmerkungen im Koalitionsvertrag; das Wort Demenz taucht dort nicht auf.

Das erste Amtsjahr der Regierungskoalition war seit Februar 2022 stark durch die Außen- und Sicherheitspolitik sowie konsekutiv die Energiepreiskrise geprägt. Dadurch ergaben sich ungeplante Gesetze und Verordnungen, s. beispielsweise den erwähnten Hilfsfonds zur Unterstützung bei gestiegenen Energiekosten für verschiedene Gesundheitseinrichtungen. Dies hat sicherlich ministerielle Kapazitäten gebunden, welche sonst für andere Vorhaben aus dem Koalitionsvertrag hätten eingesetzt werden können.

Aber auch zuvor gab es andere ministerielle Prioritäten, wie beispielsweise Maßnahmen gegen die COVID-19-Pandemie und die Einführung einer Impfpflicht, welche am 07.04.2022 keine Mehrheit im Bundestag fand [5]. Andere Umsetzungen, wie die Pflegepersonalregelung (PPR) 2.0 aus dem Krankenhauspflegeentlastungsgesetz, sind für die stationäre Krankenversorgung gedacht und kein Instrument für die Langzeitpflege, wie im Koalitionsvertrag vorgesehen. Nur zum Themenkomplex Sterbehilfe liegen bereits Gesetzentwürfe vor, wobei dieses Thema aus der letzten Legislaturperiode stammt. Beim Klimaanpassungsgesetz ist das Bundesumweltministerium führend. Bisher liegt ein Referentenentwurf vor, und eine Anhörung wurde eingeleitet. Beim geplanten Helmholtz-Zentrum für Alternsforschung ist eine Standortwahl gefallen, und die Konzeption wurde begonnen.

Für das zweite Halbjahr 2023, welches in unseren betrachteten Zeitraum nicht eingeschlossen war, sind viele weitere Gesetzesvorhaben angekündigt; kurz vor der parlamentarischen Sommerpause wurde ein Referentenentwurf des Gesundheitsversorgungsstärkungsgesetzes vorgelegt, in welchem auch Regelungen zu Community Health Nurses getroffen werden sollen [2].

## Schlussfolgerung

Der Koalitionsvertrag 2021 zwischen SPD, Grünen und FDP nimmt an mehreren Stellen Bezug zu einer älter werdenden Gesellschaft und den damit verbundenen Herausforderungen.

In Anbetracht der erwarteten starken Verschiebung der Altersverhältnisse und des erwarteten Zuwachses der Bevölkerungsanteile im Rentenalter werden allerdings wenige Lösungsvorschläge für genuin geriatrische Aspekte gemacht. Die Pflege als einer der dringlichsten Bereiche stellt hier eine Ausnahme dar. Wünschenswert wären ein erkennbares Bewusstsein sowie Lösungsvorschläge für die Defizite in der geriatrischen Rehabilitation gewesen, auch hätten geriatrische Erkrankungsbilder im Fokus der nächsten Jahre stehen können, beispielsweise zur weiteren Umsetzung der Nationalen Demenzstrategie [15].

Ob die aufgeführten Maßnahmen in den weiteren gut zwei Jahren umgesetzt werden, und welche Wirkung sie für eine ältere Bevölkerung entfalten, wird noch zu beobachten sein.
